# Venous thromboembolism and lung cancer: a review

**DOI:** 10.1186/s40248-015-0021-4

**Published:** 2015-09-15

**Authors:** Carolina Vitale, Maria D’Amato, Paolo Calabrò, Anna Agnese Stanziola, Mauro Mormile, Antonio Molino

**Affiliations:** First Division of Pneumology, High Speciality Hospital “V. Monaldi” and University “Federico II” Medical School, Naples, Italy; Department of Cardiology, High Speciality Hospital “V. Monaldi”, Second University of Naples, Naples, Italy

**Keywords:** Venous thromboembolism, Lung cancer, Non small-cell lung cancer, Small-cell lung cancer, Thromboprophylaxis

## Abstract

Venous thromboembolism (VTE) is a common complication of malignancies and epidemiological studies suggest that lung cancer belonged to the group of malignancies with the highest incidence rates of VTE. Risk factors for VTE in lung cancer patients are adenocarcinoma, NSCLC in comparison with SCLC, advanced disease, pneumonectomy, chemotherapy including antiangiogenic therapy. Other risk factors are pretreatment platelet counts and increased release of TF-positive microparticles. Elevated D-dimer levels do not necessarily indicate an increased risk of VTE but have been shown to be predictive for a worse clinical outcome in lung cancer patients. Mechanisms responsible for the increase in venous thrombosis in patients with lung cancer are not understood.

Currently no biomarker is recognized as a predictor for VTE in lung cancer patients.

Although several clinical trials have reported the efficacy of antithrombotic prophylaxis in patients with lung cancer who are receiving chemotherapy, further trials are needed to assess the clinical benefit since these patients are at an increased risk of developing a thromboembolism.

## Introduction

Thromboembolism is a well recognized complication of malignant disease and it is known that cancer patients have a higher incidence of venous thromboembolism (VTE), including pulmonary embolism (PE) and deep venous thrombosis (DVT), compared to the general population [1].

The incidence of thromboembolic disease in general population is relatively low, about 1–3 per 1.000 per year [2] while among cancer patients, the occurrence of VTE is 4–7 times higher, depending on the type and the stage of cancer [3].

It is estimated that cancer is responsible for 20 % of all cases of incident VTE [4].

Epidemiological studies suggest that lung cancer belonged to the group of malignancies with the highest incidence rates [5–9] of VTE.

The association between VTE and lung cancer has been reported more than 20 years ago [10, 11].

Lung cancer is the leading cause of cancer death in the United States for both men and women, with over 156,000 deaths in 2011 in the United States [9]. It is also one of the malignancies that are commonly associated with VTE, including PE, with reported incidence of VTE 3–13.8 % and that of PE up to 3.8 % [9, 12, 13].

In recent years, there has been increasing attention to the phenomenon of VTE in lung cancer patients and several studies have been made to better characterize it.

## Review

### Epidemiology of lung cancer-associated thrombosis

It is recognized that patients with lung cancer are at risk for development of VTE. The incidence of VTE in lung cancer patients has been described in several studies.

Chew *et al*. [14] investgated the incidence of VTE and the risk factors associated with development of VTE in a large popuation-based study of patients with non small cell (NSCLC) and small cell lung cancer (SCLC). Among 91.933 patients with newly diagnosed lung cancer, the 1-year and 2-year cumulative VTE incidence were 3.0 and 3.4 %, respectively, with a person-time rate of 7.2 events/100 patient-years during the first 6 months [14].

In a retrsospective analysis of a lung cancer cohort of 6732 patienst (control cohort had 17,284 patients), VTE occurred in 13,9 % of the lung cancer cohort and 1,4 % of the control cohort [15].

In another retrospective study of 1940 patients with diagnosis of lung cancer, thromboembolic events were documented in 9.8 % cases, venous thromboembolic complications in 78 %, arterial thromboembolic complications in 27 % cases. About venous thromboembolic complications, it was documented deep venous thrombosis in 55 % cases and pulmonary embolism in 66 % cases [16].

Recently Zhang *et al.* [17] described the high prevalence of VTE in patients with newly diagnosed lun cancer. VTE events occurred in 89 (13,2 %) of the 673 patients enrolled in the study. Forty-two (6.2 %) patients developed lower extremity deep vein thrombosis (DVT) alone, 33 (4.9 %) patients developed pulmonary embolism (PE) and 14 (2,1 %) patients developed bth DVT and PE [17].

The incidence of thrombotic event was evaluated in a group of 950 patients with lung cancer by Kadlec *et al.* [18]. 91 (8,4 %) thromboembolic events were observed (34 cases of pulmonary embolism, 58 cases of deep venous thrombosis and 13 cases with simultaneous occurrence of both disease) [18]. The most common sources of venous thrombosis were low limbs (49,74 %) and superior vena cava (5,11 %); less frequent was thrombosis associated with implantable ports and venous catheters (3,6 %) [18].

In a retrospective review of lung cancer patients Alexander *et al.* reported that the incidence of thromboembolism was 10.8 % after a medin follow up of 10 months [19].

An unclear proportion of VTE events in lung cancer patients is incidentally discovered.

White *et al.* [20] identified incident VTE events within 1 year before the cancer diagnosis date, by using registry data. Among 528 693 cancer cases, the incidence of preceding VTE was increased over that expected in the year preceding the diagnosis of cancer, but in particular only during the 4-month period immediately preceding tha cancer diagnosis date (*p* <0.001). Almost all of these unexpected VTE cases were significantly associated with a diagnosis of metastatic-stage cancer [20].

In a more recent study, it was observed among 207 subjects with lung cancer that one-third of VTE events was incidentally discovered [21].

Calleias *et al.* [22] have evaluated the prevalence of incidental VTE detected by PET-CT among cancer patients in a retrospective study. The prevalence of incidental VTE detected by PET-CT was 1.4 % [22]. Patients with genitourinary malignancies, colorectal cancer and lung cancer had the highest rates of incidental VTE at PET-CT [22].

## Pathogenesis of VTE in cancer: evidences for lung cancer

Mechanisms responsible for the increase in venous thrombosis in patients with cancer are not understood. Rather than one unifying mechanism, the etiology is likely multifactorial with different factors assuming lesser or greater degrees of importance depending on the clinical setting.

Over the years, researchers have investigated intrinsic properties of tumor cells that lead to a prothrombotic state. It is recognized that tumors shed membrane particles that contain procoagulant activity including tissue factor and membrane lipids that propogate the coagulation response [23, 24].

Several studies have focused on the role of Tissue Factor (TF) in the pathophysiology of cancer associated thrombosis.

Tissue factor (TF) is the physiologic initiator of coagulation and is commonly expressed in a variety of malignancies [25, 26]. Overexpression of TF in tumor cells or elevated TF levels in association with microparticles in the systemic circulation may contribute to systemic hypercoagulability [27]. Tumor cells express TF and spontaneously release TF-positive microparticles (MPs) into the blood. MPs are small membrane vesicles that are highly procoagulant. Several studies have shown that increased levels of TF-positive MPs correlate with venous thrombosis in patients with cancer.

In the past, some studies demonstrated the expression of TF by lung cancer cells [28, 29].

Sawada *et al.* [30] have previously demonstrated increased TF expression in malignant cells from patients with lung cancer. Remarkably, in their study, TF expression levels correlated with the presence of metastatic tumors. Goldin-Lang *et al.* [31] also demonstrated increased TF expression in NSCLC, although no direct correlation between disease stage and TF expression levels was detected.

More recently the expression TF protein and/or mRNA have been documented in malignant cells or tissue in NSCLC [32].

Sato *et al.* [33] described a case of Trousseau’s syndrome that shows the possible contribution of TF in the pathogenesis of DVT/PE in patients with lung cancer. They observed a marked increase in plasma TF level, together with positive staining of cancer cells with monoclonal anti-TF antibody. Therefore tissue factor (TF) originating from lung adenocarcinoma appeared responsible for recurrent deep vein thrombosis (DVT) and pulmonary thromboembolism (PE) [33].

Similarly, Del Conde *et al.* [34] reported a higher level of plasma TF in a patient with a giant-cell lung carcinoma with a severe form of Trousseau’s syndrome. In this case was observed a 41-fold higher concentration of plasma TF as compared with the mean plasma TF concentration examined in 16 normal individuals [34].

## Risk factors of VTE in patients with lung cancer

Cancer increases the risk of thromboembolic events. Risk factors, such as age, gender, bed-rest, venous catheters, surgery, chemotherapy with or without adjuvant hormone therapy, radiotherapy and infections, also increase the risk of thrombosis in cancer patients.

Rates of VTE vary substantially between cancer patients, considerably depending on clinical factors, the most important being tumor type and stage.

In this review we have focused on studies which investigated factors could be identified as predictors of vascular events in lung cancer and their prognostic significance.

### Haemostatic parameters

Thrombocytosis can result in thrombosis and is frequently observed in patients with malignancies The mechanism underlying development of thrombocytosis in lung cancer patients remains unclear; one of possible mechanisms might be tumor-associated elevation of bone marrow-stimulating cytokines such as interleukin (IL)-6, IL-1 and macrophage colony stimulating factor (M-CSF). These cytokines could exhibit thrombocytosis. Incidence and prognostic significance of thrombocytosis in patients with lung cancer has been investgated in some studies [35].

Pedersen and Milman [36] examined data on platelet counts obtained in a large population (1178 patients) of patients with primary lung cancer. A high prevalence (32 %) of elevated platelet counts was observed in patients with lung cancer. The frequency of thromboembolism in patients with thrombocytosis was not statistically different from the frequency found in patients with normal platelet counts so, according the authors, thrombocytosis should not be considered as a risk factor predisposing to thromboembolism in cancer patients [36]. Thrombocytosis was associated with the tumour load according to the disease stage. Surgical debulking of the tumour resulted in significantly reduced platelet counts. Respect to survival, patients with thrombocytosis had a significantly poorer survival than patients with normal platelet counts. According to the results of this study, thrombocytosis is an indipendent prognostic factor of survival in patients with primary lung cancer [36].

Tomita *et al.* [35] evaluated the prognostic effect of thrombocytosis in patients with resectable NSCLC. 240 consecutive NSCLC patients, who received surgical resection, were reviewed retrospectively and the survival impact of preoperative platelet count was investigated. The 5-year survival of patients with and without thrombocytosis was 28,87 and 63,73 %, respectively (*p* < 0.0001). Therefore preoperative platelet count was a prognostic factor for resectable NSCLC patients [35].

These findings are supported by a more recent study [37] that assessed the prognostic value of thrombocytosis and its relation with patients undergoing surgery for lung cancer. Thrombocytosis appeared most frequently in patients with squamous cell lung cancer and among smokers. The presence of perioperative thrombocytosis in patients undergoing surgery had a negative prognostic value (the overall 5-year survival was worse in patients with thrombocytosis (*p* < 0,001) [37].

Recently Kadlec *et al.* [18] observed in a large cohort of lung cancer that patients with thromboembolic events showed significantly higher levels of platelet count at the time of diagnosis of lung cancer and a platelet count above 330.5 × 10^9^ was associated with OR for major thromboembolic events was 3.66 (2.25–5.96) — a statistically significant value. Thrombocytosis was found in patients with advance disease, more commonly in men, with no apparent correlation with the clinical stage of the disease [38].

On the contrary, Demirci *et al.* in a restrospective study of consecutive 281 patients with lung cancer did not observe a significant correlation between vascular events and thrombocytosis [39].

Plasma levels of D-dimer are elevated in cancer patients. Activation of the extrinsic coagulation system and the fibrinolytic cascade within a tumour is thought to be related with growth, invasion and metastasis. Although high D-dimer was described as a predictor of VTE in earlier works, it is now recognized that it does not necessarily indicate an increased risk of VTE in cancer patients [18].

Several studies have demonstrated that elevated D-dimer levels are associated with a poor prognosis in cancer patients. In patients with both non-small cell and small cell lung cancer, increased plasma D-dimer levels were shown to be predictive for a worse clinical outcome [40].

In addition in cancer lung patients it has been shown that D-Dimer plasma levels decrease or increase after response and progressive disease, respectively, and can act as a predictive factor of the evolution of the disease [41].

Recently Ferroni *et al.* [42] hypothesized that the use of a novel high-sensitive D-dimer determination could predict chemotherapy associated VTE in lung cancer patients with intermediate risk, according to Khorana’s assesment model [43]. Overall 108 lung cancer patients at the start of a new platinum-based chemotherapy were evaluated. A cutoff level of 1500 was obtained by statistical analysis; it resulted in a sensitivity of 81 %, a specificity of 69 %, a positive predictive value of 31 %, a negative predictive value of 96 % and an accuracy of 70 %. Patients with HS-D-dimer levels above the cutoff had a worse VTE- free survival (60 %) compared with those with levels below the cut-off (95 %, *p* = 0.0001) [42]. The use of High Sensitive D-dimer determination prior to chemotherapy may help to identify cancer patients with an intermediate risk of VTE who could benefit from thromboprophylaxis [42].

With regard to other hemostatic factors, there are different results in the literature.

Gabazza *et al.* [11] found significantly higher levels of thrombin-antithrombin complexes III, D-dimer, and plasmin-alpha 1-antiplasmin complexes in lung cancer patients, suggesting an increased activation of coagulation and fibrinolysis.

In a prospective study Zecchina *et al.* [44] investigated the activation of the hemostatic factors in 45 patients undergoing chemotherapy for lung cancer. They reported no significant changes in such parameters as D-dimer, fibrinogen, and activated protein C, however found a significant increase in platelet count 21 days after the administration of chemotherapy, correlating with the occurrence of VTE.

Elevated levels of procoagulant factors, such as lupus anticoagulant, anticardiolipin antibodies to factor VIII, and certain cytokines such as interleukin-6 and tumor necrosis factor, have also been identified as increased risk factors for VTE in patients with lung cancer [28, 29].

Recently Kadlec *et al.* in a study population of 950 lung cancer patients did not find significantly higher medians of coagulation parameters (D-dimer, fibrinogen, thrombin time and aPTT) [18].

### Hystological types of lung cancer

Over the years several studies investigated the VTE risk in different hystological types of lung cancer.

In the past, autopsy and retrospective studies indicated that various adenocarcinomas are most strongly associated with VTE [45, 46]. This has led to the widespread belief that mucin-producing adenocarcinomas are indeed the most common tumours associated with VTE. This notion was supported by the findings of several other studies [14, 15, 47]. Blom *et al.* [47] investigated thrombotic risk in 537 NSCLC patients and observed that the risk of VTE was 20-fold higher than in the general population (standardized morbidity ratio: 20.0 (14.6–27.4). Patients with adenocarcinoma of the lung had three-fold higher risk (incidence 66.7 per 1000 years) than patients with squamous cell carcinoma of the lung (incidence: 21.2 per 1000 years). In adeno and squamous carcinoma together, they observed 39 VTE events over 879 years of follow up for an overall incidence of VTE of 44.4 per 1000 person years [47].

In a restrospective analisys of a large cohort of patients with NSCLC and SCLC, was observed that adenocarcinoma histology (HR = 1.9 vs. squamous cell, 95 % CI = 1.7–2.1) and advancing cancer stage (HR = 4.0 for metastatic vs. local disease, 95 % CI = 3.4–4.6) were significant predictors of developing VTE within 1 years of NSCLC diagnosis [14].

Recently Kadlec *et al.* and Lee [18, 48] identified adenocarcinoma histology as a risk factor for development of VTE.

Tagalakis *et al.* [13] described a high incidence (13,6 %) of deep vein thrombisis in a cohort of 493 patients with NSCLC.

In a retrospective analysis of lung cancer patients, Alexander *et al.* [19] observed, that thromboembolism occurred equally in patients with non-small cell and small cell lung cancer (10.8 and 10.5 % respectively), and more frequently among patients with adenocarcinoma compared to squamous cell carcinoma (14.7 and 5.3 % respectively) [19].

Differently not a significant correlation between vascular events and histological type, or TNM stage was observed in a retrospective study by Demirci *et al.* [39].

VTE was a significant predictor of death within 2 years for both NSCLC and SCLC, HR = 2.3, 95 % CI = 2.2–2.4, and HR = 1.5, 95 % CI = 1.3–1.7, respectively [14].

### Cancer therapy

Cancer therapy itself has been shown to increase the risk of VTE, whether it be chemotherapy, antiangiogenic therapy, or hormonal therapy.

Combined chemotherapy (in combination with radiotherapy) is the current standard treatment for advanced stage NSCLC as well as in the treatment of SCLC patients and VTE is a well known complication of anticancer therapy [49, 50].

How chemiotherapy contributes to VTE risk has not to our knowledge been elucidated completely but likely involves a combination of direct endothelial damage and down regolation of endogenous anticoagulants [51].

In an observational study by Blom *et al.* [47], the overall incidence of VTE in an ambulatory population with lung cancer starting a new chemotherapy was 1.93 % over a median follow up period of 2.4 months. The rate of VTE observed in this study (0,8 % per month) was substantially in excess of the estimated rate of approximately 0.04 % per month for the entire cancer population. The data suggest that chemotherapy is associated with three-fold increased risk for VTE over and above this already elevated rate, with further increases with time on chemotherapy [47].

Khorana *et al.* [52] analysed data from a prospective multicentre study in 3003 patients treated with at least one cycle of chemotherapy. They reported an odds ratio for VTE of 1.86 % (95 % CI, 0.67-3.38) in lung cancer patients [52].

The results of this study are supported by Hicks *et al.* [53] that have conducted a pooled analysis of three clinical trials. They observed that the risk of VTE is increased in patients with NSCLC at all stages who receive chemotherapy, but not erlotinib or BMS-275291, whether it is given as postoperative adjuvant therapy or fort he treatment of advanced disease [53].

Numico *et al.* [54] prospectively assessed the occurrence of VTE in patients with NSCLC who were treated consecutively with cis-platin and gemcitabine. They observed 22 VTEs in 19 of 108 stage II-IV NSCLC patients who underwent chemotherapy (17,6 %; 95 % CI 10.3–24.8) [54].

Recently a retrospective study of lung cancer patients showed that chemotherapy-treated patients experienced thromboembolism more often than patients who did not receive chemotherapy (HR 5.7 95 % CI 2.2–14.8) [19].

Among lung cancer patients receiving chemotherapy, it was described that the majority of VTE events occurring within 6 months of initiation of chemotherapy [55]. The presence of a VTE event is significantly associated with an increased risk of mortality [55].

With regard to antiangiogenic chemotherapy, researchers have focused on neoangiogenesis inhibition with VEGF inhibitor bevacizumab.

While some trials suggested that bevacizumab may be associated with increased risk for VTE [56], others indicate that bevacizumab is not associated with significant elevation risk in the context of contemporary chemotherapy regimens [57].

Scappaticci *et al.* [58] conducted a post hoc analysis of pooled data from randomized controlled trials evaluating combination treatment with bevacizuamb and chemotherapy vs chemotherapy alone in 1745 patients with colorectal, breast, or non small cell lung cancer. Compared with chemotherapy alone, bevacizumab was associated with a 2-fold increase in arterial thromboembolic events (*p* = 0.031) but was not found to be associated with an increased risk of venous thromboembolic events [58].

These data are in contrast to a recent systematic review and meta analysis by Nalluri *et al.* [59]; results indicated that bevacizumab was associated with an increased risk of VTE, with a relative risk (RR) of 1.33 (95 % CI, 1.13–1.56, *p* < 0.001) compared with controls [59].

Antiangiogenic agents also contribute to thrombosis, perhaps through endothelial cell and platelet activation [27].

Postoperative use of angiogenesis drugs EGFR-TKI application were high risk factors for VTE in lung cancer patients [60].

With regard to metalloproteinase inhibitor prinomastat there are divergent data.

According to Behrendt *et al.* [61], the use of the metalloproteinase inhibitor prinomastat in combination with chemotherapy, but not alone, resulted in approximately doubling oft he risk of VTE in advanced NSCLC patients [61].

In contrast to these data in a more recent study the addition of another metalloproteinase inhibitor BMS-275291 to chemotherapy in patients with advanced NSCLC did not increase the risk [53].

The addition of aprinocarsen, a protein kinase C- alfa antisense oligonucleotide, to standard chemotherapy in patients with advanced stage NSCLC, significantly increased thromboembolism [62].

### Surgery

It is known that patients undergoing surgery for cancer have a higher risk of postoperative DVT, despite thrombosis prophylaxis [63], than those having surgery for non malignant diseases [64]. Cancer patients have at least twice the risk of postoperative DVT and are known to have a more than three-fold increased risk of fatal pulmonary embolism than non cancer patients undergoing similare procedures [65].

Among patients who undergo surgical resection due to oncological processes, VTE is considered a major cause of mortality and may serve as an important predictor of survival.

With regard to lung cancer, Ziomek *et al.* [66] analyzed 77 patients who underwent lung resection surgery for lung cancer. They found that 20 patients (26 %) had a thromboembolic episode in the postoperative period (15 cases of DVT and 5 cases of PE) [66].

Lyman *et al.* [67] established that the risk of having a fatal PE after surgery is three times higher than in patients undergoing the same surgery for benign causes.

Pneumonectomy has been associated with a higher risk of VTE than lobectomy for stage I and II lung cancer, due to greater activation of coagulation, which appears from the seventh postoperative day [68].

Mason *et al.* [69] described a higher incidence (7.4 %) of postoperative VTE after pneumonectomy for malignancies as compared with that reported in older literature, with a peak incidence within 7 days after the operation.

Yang *et al.* [60] explored clinical risk factors for VTE in postoperative lung cancer patients. They described that the highest incidence of VTE is within 1 month after lung cancer surgery and high risk factors for VTE include incomplete surgical resection, postoperative use of anti-angiogenesis drugs, EGFR-TKI application and an increase in preoperative D-dimer level [60].

Recently Kadlec *et al.* [18] reported in a study population of 950 patients with lung cancer, that patients undergoing surgery had an incidence of 17.6 % of VTE (11 and 48.4 % for those undergoing radiotherapy and chemotherapy, respectively).

Differently Dentali *et al.* [70] have observed a low incidence of VTE in lung cancer patients undergoing surgery procedures. They described, in a series of 693 patients who underwent thoracotomy for lung cancer under an antithrombotic prophylaxis protocol, an incidence of VTE and PE of 1.7 and 1.3 %, respectively, with 0.6 % mortality attributed to PE [70].

Gomez-Hernandez *et al.* [71] have found a low prevalence of VTE in a retrospective study of 6004 patients undergoing elective thoracic surgery procedures. Among surgical procedures lobectomy and pneumonectomy were the most common and lung cancer was the main diagnosis (2245 cases). The prevalence of VTE was 0,18 % [71]. According to Gomez-Hernandez *et al.*, this low prevalence may be attributable to the use of thromboprophylaxis protocols, which include the use of anticoagulant drugs and mechanical measures (intermittent compression systems and elastic stockings) and early ambulation [71].

A recent published review by Christensen *et al.* [72] analysed 19 studies with a total of 10,660 patients with primary lung cancer undergoing operations with curative intent. The mean risk of VTE was estimated at 2.0 % (range, 0.2–19 %), with a mean observation period of 16 months (range, 0.1–22). The risk of VTE seems to occur predominantly within the initial postoperative period, and subsequently the risk falls [72].

With respect to mortality, it was described that pulmonary embolism is the second cause of mortality after pneumonectomy for a malignancy and such patients have the highest risk of dying from pulmonary embolism [73]. Weder *et al.* [74] in their series of 176 patients who underwent pneumonectomy due to lung cancer after neoadjuvant chemotherapy, described a mortality of 3 %; half were attributed to pulmonary thromboembolic complications [74].

Interestingly, mortality due to VTE has been reported to occur predominantly in patients with squamous cell lung carcinoma following surgery [73].

## VTE prophylaxis and treatment in cancer patients: evidences for lung cancer

In cancer patients the risk of VTE is particularly high in association with surgery for cancer and chemotherapy.

Haas *et al.* [75] investigated the efficacy of low-molecular-weight heparin (certoparin 3000 IU once daily for 6 months) in the prevention of venous thromboembolism among ambulatory cancer patients. In two double blind studies, they analyzed patients with metastatic breast cancer (TOPIC −1) or patients with III/IV stage NSCLC (TOPIC-2). A post hoc analysis showed certoparin significantly reduced VTE in stage IV lung carcinoma (3.5 % vs 10.2 %; *P* = .032) without increased bleeding [75].

Zhang *et al.* [76] have recently publicated a systematic review and meta-analysis about the efficacy and safety of adjunctive anticoagulation in patients with lung cancer without indication for anticoagulants. The analysis of 2185 patients showed that anticoagulation reduced the incidence of VTE (RR = 0.55, 95 % CI 0.31–0.97; *p* = 0.04) and thromboembolic events (RR = 0.48, 95 % CI 0.28–0.82; *p* = 0.008). Furthermore anticoagulation showed significant improvement in survival at 1 year (RR 1.18, 95 % CI 1.06–1.32; *p* = 0.004) and at 2 years (RR 1.27, 95 % CI 1.04–1.56; *p* = 0.02), especially for patients with SCLC and prolonged life expectancy [76]. Between subcutaneus heparin and vitamin K antagonist, heparin was associated with a lower risk of bleeding [76].

In a retrospective analysis of lung cancer patients, patients receiving chemotherapy experienced thromboembolism more often than patients who did not receive chemotherapy (HR 5.7 95 % CI 2.2–14.8) [19]. Pharmacological thromboprophylaxis was not routinely or systematically prescribed for lung cancer outpatients during any treatment phase [19]. The majority (83 %) of thromboembolic events occurred in the ambulatory care setting. It was stressed that thromboprophylaxis guidelines should be developed for the ambulatory care setting [19].

Recently the neutrophil-lymphocyte ratio (NLR) was identified as a potentially useful marker for predicting clinical outcome in patients with anticoagulation for VTE. The study has demonstrated that NLR at the time of VTE diagnosis could be a useful biomarker for predicting the response and prognosis following anticoagulation in patients with lung cancer and VTE [77].

The CARMEN study is an interesting french observational study, in which the researchers evaluated the adhesion to guidelines for treatment of VTE in hospitalized patients [78].

Among cancer patients, 64 % had metastatic disease. Cancer sites were gastro-intestinal (25 %), gynecologic (23 %), pulmonary (21 %), hematological (14 %), urologic (10 %), or other (8 %) [78].

It was observed that overall adhesion to guidelines was present in 59 % of patients. During initial treatment, adhesion was high but dropped during the long-term mantenance. Lung and hematological malignancies were significantly associated with the highest and lowest rates of adhesion [78].

Conversely Alexander *et al.* [79] made a survey among clinicians regarding appropriate thromboprophylaxis for patients with lung cancer in order to identify variation in practice and/or divergence from evidence-based clinical practice guidelines. Clinicians consistently identified patients with lung cancer as having a high thromboembolic risk in both ambulatory and surgical settings, but with differences in recommendations and variation in practice [79].

## Conclusions

Venous thromboembolism, including pulmonary embolism and deep venous thrombosis, is a common occurrence within the cancer population. Lung cancer is one of the most common cancer in western countries with reported high incidence of VTE. VTE in hospitalized lung cancer patients is associated with longer length of stay, higher inpatient mortality rates, increased cost and greater disability upon discharge compared to other inpatient lung cancer patients [80]. Risk factors for VTE in lung cancer patients are adenocarcinoma, NSCLC in comparison with SCLC, advanced disease, pneumonectomy, chemotherapy including antiangiogenic therapy. Other risk factors are pretreatment platelet counts and increased release of TF-positive microparticles. Elevated D-dimer levels do not necessarily indicate an increased risk of VTE but have been shown to be predictive for a worse clinical outcome in lung cancer patients.

Currently no biomarker is recognized as a predictor for VTE in lung cancer patients.

Although several clinical trials have reported the efficacy of antithrombotic prophylaxis in patients with lung cancer who are receiving chemotherapy, further trials are needed to assess the clinical benefit since these patients are at an increased risk of developing a thromboembolism.

## Abbreviations

CI: Confidence interval
DVT: Deep venous thrombosis
EGFR: Epidermal growth factor receptor
HR: Hazard ratio
LMWH: Low molecular weight heparin
NLR: Neutrophil-lymphocyte ratio
NSCLC: Non-small-cell lung cancer
OR: Odds ratio
PE: Pulmonary embolism
RR: Relative risk
SCLC: Small-cell lung cancer
TF: Tissue factor
VEGF: Vascular endothelial growth factor
VTE: Venous thromboembolism
